# Why do some physicians in Portuguese-speaking African countries work exclusively for the private sector? Findings from a mixed-methods study

**DOI:** 10.1186/1478-4491-12-51

**Published:** 2014-09-11

**Authors:** Giuliano Russo, Bruno de Sousa, Mohsin Sidat, Paulo Ferrinho, Gilles Dussault

**Affiliations:** Department of International Health and Biostatistics, Instituto de Higiene e Medicina Tropical, Nova University of Lisbon, Rua da Junqueira 100, Lisbon, Portugal; Centro de Malária e outras Doenças Tropicais, Instiuto de Higiene e Medicina Tropical, Lisbon, Portugal; Department of Psychology and Education Sciences, University of Coimbra, Coimbra, Portugal; Faculty of Medicine, Eduardo Mondlane University, Maputo, Mozambique

**Keywords:** Human resources for health in low- and middle-income countries, Health systems in Portuguese-speaking African countries, Private health sector in Africa, Private sector physicians

## Abstract

**Background:**

Despite the growing interest in the private health sector in low- and middle-income countries, little is known about physicians working outside the public sector. The present work adopts a mixed-methods approach to explore characteristics, working patterns, choices, and motivations of the physicians working exclusively for the private sector in the capital cities of Cape Verde, Guinea Bissau, and Mozambique. The paper’s objective is to contribute to the understanding of such physicians, ultimately informing the policies regulating the medical profession in low- and middle-income countries.

**Methods:**

The qualitative part of the study involved 48 interviews with physicians and health policy-makers and aimed at understanding the practice in the three locations. The quantitative study included a survey of 329 physicians, and multivariate analysis was conducted to analyse characteristics, time allocation, earnings, and motivations of those physicians working only for the private sector, in comparison to their public sector-only and dual practice peers.

**Results:**

Our findings showed that only a limited proportion of physicians in the three locations work exclusively for the private sector (11.2%), with members of this group being older than those practicing only in the public or in both sectors. They were found to work fewer hours per week (49 hours) than their public (56 hours) and dual practice peers (62 hours) (*P* <0.001 and *P* = 0.011, respectively). Their median earnings were USD 4,405 per month, with substantial variations across the three locations. Statistically significant differences were found with the earnings of public-only physicians (*P* <0.001), but not with those of the dual practice group (*P* = 0.340). The qualitative data from the interviews showed private-only physicians’ preference for an independent and more flexible work modality, and this was quoted as a determining factor for their choice of sector. This group appears to include those working in the more informal sector, and those who decided to leave the civil service following a disagreement with the public employer.

**Conclusions:**

The study shows the importance of understanding the relation between health professionals’ characteristics, motivations, and their engagement with the private sector to develop effective policies to regulate the profession. This may ultimately contribute to achieving universal access to medical services in low- and middle-income countries.

**Electronic supplementary material:**

The online version of this article (doi:10.1186/1478-4491-12-51) contains supplementary material, which is available to authorized users.

## Background

The private sector in low- and middle-income countries (LMICs) is attracting growing interest as a valid instrument to reach the poor [1, 2]. In some sub-Saharan African countries the emergence of private health services in urban areas is a relatively recent phenomenon [3], placing pressure on the public sector to compete for scarce health workers [4], and creating opportunities for multiple job-holding [5]. The private health sector in LMICs can be very diverse and include for-profit and not-for-profit providers, non-governmental and faith-based organisations (NGOs), groups and individual providers, and even shopkeepers [2]; some of these operate within the formal regulated healthcare system, and others on its margins [6].

The literature on qualified private sector physicians (PSP) in LMICs mainly focuses on the description and quality of their practice [7, 8] and it rarely distinguishes between those who work exclusively for the private and those engaging simultaneously with private and public sector activities, the so-called dual practitioners. Some scholars have analysed PSPs’ motivations to identify more effective public sector retention strategies [9, 10]. A study of PSPs’ work satisfaction in South Africa found that they are overall content with their work conditions, but dissatisfied with the high proportion of uninsured patients, low earnings, high administrative burden, and with the associated pressure to reduce costs [11]. Lonnroth et al. [12] identified several obstacles to pursue private employment among PSPs in Vietnam such as the burden of initial investment, gender discrimination, and regulatory barriers. Malik et al. [13] showed that PSP motivational determinants differ considerably among levels of care, sectors, and gender in Lahore, Pakistan. Topping up the public salary to meet the cost of living and supporting the extended family are found to be main reasons for engaging with the private sector in Portuguese-speaking African countries [14]. Russo et al. [15] argue that physicians’ involvement in the private sector in LMICs is more common than generally assumed, as multiple forms of private practice can be found even within public facilities.

Comprehensive research exists from high-income countries on physicians’ motivations to engage in private sector practice. These include increasing earning opportunities and having an entrepreneurial attitude [16], as well as having a preference for shorter working hours [17]. These findings are in line with the work by Midttun [18], who shows that among Norwegian specialists, a high valuation of professional values push them towards public employment [19]. Financial gains and opportunities for out-of-hours activities are the motivations reported by British general practitioners for working in the private sector [20]. In the Netherlands, physicians’ gender and preference for shorter working hours are found to affect their choice of practice sector [17].

In Cape Verde, Guinea Bissau, and Mozambique private health care was practiced and regulated during the Portuguese colonial period, but was momentarily discontinued by the socialist regimes after independence. Both Guinea Bissau and Mozambique experienced civil war, which substantially weakened their health systems. Mozambique has known peace since 1992, while Guinea Bissau is still marred by violent political confrontations [21]. In contrast, Cape Verde operated a peaceful post-independence democratic transition, and has recently achieved middle-income country status [22]. Physician private practices in Cape Verde are regulated, including within public facilities [23]. In Mozambique, private health care services regulation is still under development, with ‘special’ private services being offered within public sector facilities [24]. In Guinea Bissau, private sector regulation is still underdeveloped [25], and illegal charges are reported to be ubiquitous in public facilities [26]. In the three countries, the opportunities for private sector health care practice are concentrated in their capital cities, Praia, Bissau, and Maputo [15].

This paper draws from physicians’ interviews and survey data collected in the three capital cities, with the aim of deepening the current understanding of those physicians working exclusively for the private sector in LMICs. Our hypothesis is that such motivations can be adequately explored by contrasting experiences, working patterns, choice of practice sector, and personal characteristics of the physicians from the three geographical settings. The ultimate objective of the paper is to provide an insight for the regulation of the profession and inform the elaboration of policies to increase population access to medical services in LMICs.

## Methods

This study is based on the secondary analysis of interviews and survey data collected between January and May 2012 in a related study of physician dual practice in Praia (Cape Verde), Bissau (Guinea Bissau), and Maputo (Mozambique) [15]. The present study focuses on the characteristics, working patterns, and motivations of those physicians working exclusively for the private sector, in comparison to their dual practice and public sector-only peers. The three capital cities were selected because of their similarities in terms of cultural heritage and health systems, despite the different stages of development of their private health sector. The ‘private-only’ group included qualified physicians registered with the National Medical Associations and working exclusively for the for-profit private sector or for NGOs and religious medical organizations; the ‘public-only’ group consisted of those working exclusively for the public; and the ‘dual practice’ group consisted of those working simultaneously in both formal sectors. Other forms of physician employment, such as in health policy-making or outside the health sector, were not considered.

In a first stage, 48 key informants were interviewed; these were policy-makers and physicians practicing in the public and private sector in the three capital cities. The interview guide was elaborated on the basis of the literature on physician economic behaviour, motivation, and coping strategies, and explored physicians’ working time allocation across jobs, motivation and incentives, and perceptions of regulation of the profession. Qualitative findings from the interviews were used to design the survey questionnaire used in the second stage of the study.

For the survey, 329 physicians registered with the National Medical Associations were randomly selected, representing 52% of the overall physician population in the three locations. The full survey’s data collection methodology is described elsewhere [15]. Physicians were asked to describe their professional activities in the previous working week in terms of hours and medical acts performed. To calculate earnings, physicians were asked about their monthly net salary in the public sector; to calculate private sector earnings, we multiplied the medical acts declared by their average private sector prices (see Survey Questionnaire in Additional file 1). Since it emerged that a variable administrative fee is retained by the clinic where the medical act takes place, we made the simplifying assumption that physicians only earn 60% of the private service revenues. Earnings were calculated in local currencies; to make comparisons possible across the three cities, we converted them to United Stated Dollars (USD) at exchange rates and purchasing parity power dollars [27]. We consider the limitations of such methodological choices in the discussion.

### Analysis

Interviews were audio recorded, transcribed, manually coded, and analysed for contents in Portuguese. The coding tree focused on the identification of key themes and categories on three main areas, namely: (a) characteristics of the market for private medical services, (b) current legislation, history, and perceptions of private sector regulation, and (c) physicians’ motivations and incentives to work in the private and/or public sectors.

Descriptive and multivariate analysis was carried out in IBM SPSS Statistics – Version 20. For the descriptive analysis, proportion Z-tests with Bonferroni adjusted *P* values were performed in order to determine any possible differences among the three groups of doctors’ employment considered. In order to analyse the independence of variables, χ^2^ tests were performed and, when the hypothesis of independence was rejected, the corresponding residual analysis was carried out. When conditions to apply the χ^2^ test failed, Fisher’s exact test results were presented. To overcome the failure of normality (Kolmogorov-Smirnov and Shapiro-Wilk tests) and homogeneity of variances (Levene’s test) necessary for the application of the *t*-test when comparing quantitative variables between the different groups, the non-parametric Kruskal-Wallis test was applied and, when appropriate, multiple comparisons were presented. All statistical tests were performed considering a 5% significance level.

## Results

### Characteristics of private sector physicians (PSPs)

PSPs were found to be a minority in the three locations. The survey sample of physicians providing clinical services included 37 who declared that they worked exclusively in the private sector (11.2%), 42.2% worked only in the public sector (139), and 46.5% engaged in both sectors (153) (Table 1). In the private-only group there were fewer men (48.6%) and married physicians (59.5%) than in the other two groups, with the majority holding a specialization (73%). Proportion Z-tests with Bonferroni-adjusted *P* values did not highlight any statistically significant differences between the three types of physicians with regards to sex, marital status, and specialization.

**Table 1:** **Physicians’ personal characteristics, functions, and modality of work by type of employment**

	Type of employment							
		Private		Public		Dual practice		Total
	Count	%	Count	%	Count	%	Count	%
City								
Praia	14	12.8	39	35.80	56	51.40	109	100.00
Maputo	8	6.40	53	42.40	64	51.20	125	100.00
Bissau	15	15.80	47	49.50	33	34.70	95	100.00
Total	37	11.20	139	42.20	153	46.50	329	100.00
Sex (M)*	18	48.60a	74	53.20a	85	55.60a	177	53.80
Civil status (M)*	22	59.50a	100	72.50a	118	77.10a	240	73.20
With a specialization*	27	73.00a	72	51.80ab	115	75.20b	214	65.00
Function								
As a clinician*	28	75.70a	–	–	148	96.70b	176	92.60
As a manager*	6	16.20a	–	–	13	8.50a	19	10.00
As a consultant*	2	5.40a	–	–	3	1.30a	5	2.60
Other*	2	5.40a	–	–	3	1.30a	9	4.70
Modality of work								
Own private practice*	10	27.00a	–	–	32	20.90a	42	22.10
Private practice owned by colleagues*	2	5.40a	–	–	34	22.20b	36	18.90
Private clinic*	15	40.50a	–	–	82	53.60a	97	51.10
Private hospital*	4	10.80a	–	–	14	9.20a	18	9.50
House visits*	1	2.70a	–	–	3	2.00a	4	2.10
Other*	7	18.90a	–	–	13	8.50b	20	10.50
Characteristics	**Median**	**SD**	**Median**	**SD**	**Median**	**SD**	**Median**	**SD**
Age	51	13.68	42	9.74	45	9.51	45	10.24
Number of dependents	2	3.396	3	3.814	3	3.087	3	3.473
Years of work as MD in the capital city	12	9.37	7	8.933	11	8.742	9	9.016

Note: Totals do not add up to 100%, as functions and modalities of work are not mutually exclusive. *Cells in the same row not sharing the same subscript indicate proportion differences significant at 5% level in a two-sided test of equality for column proportions.

Location, age, and holding a specialization were found to be determining factors in physicians’ selection of practice sector. When looking for association between variables, χ^2^ tests rejected the null hypothesis of no association between the type of employment and the variables city (*P* = 0.029) and specialization (Yes/No) (*P* <0.001). Kruskal-Wallis test results showed statistically significant differences in median ages between physicians in the private sector (51 years) and those in the public sector (43 years) with a *P* value of 0.034, but not among PSPs and dual practitioners, or between these latter and those from the public sector, with *P* values equal to 0.488 and 0.209, respectively. Similarly, only the differences in the median number of years of practice as medical doctors between the dual practitioners (12 years) and the public sector (7 years) were statistically significant (*P* = 0.001). For the multiple comparisons regarding the number of dependents among the three groups, no statistically significant differences were found (all *P* values greater than 0.05).

### Work modalities and earnings

A greater proportion of ‘private-only’ physicians were found to work for their own practice compared to dual practitioners (27% vs. 20.9%), while a higher percentage of the latter worked for a practice owned by a colleague (22.2% vs. 5.4%). Working in a private clinic was the most common form of employment in both private and dual practice sectors (40.5% and 53.6%, respectively). Proportion Z-tests only showed statistically significant differences between private and dual practitioners when working for private practices owned by colleagues and other work place (Table 1).

Nine out of ten physicians in our survey sample were found to engage in clinical private practice, either exclusively or as dual practitioners; twice as many PSPs reported engaging in managerial functions (16.2%) than their dual practice peers (Table 1). Statistically significant differences were only found between private-only and dual practitioners when working as clinicians (Table 1).

**Figure 1:**
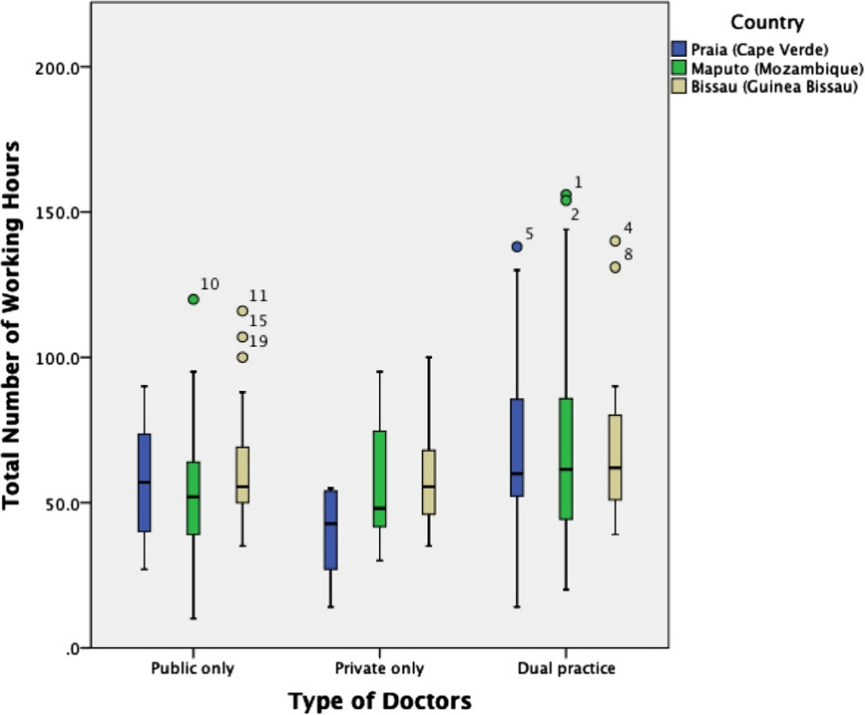
**Mean number of weekly hours worked per location and group of physicians.**

PSPs were found to work fewer hours than their peers. The median amount of hours worked per week by private-only physicians in our survey sample was 49 hours, 56 hours for public-only physicians, and 62 hours for dual practitioners (Figure 1). Private-only physicians in Praia reported working the least hours per week (42 hours), with dual practitioners in Bissau and Maputo reporting the most (62 hours) (Table 2). The Kruskal-Wallis test rejected the hypothesis of equal median values for the three groups (*P* <0.001). There were statistically significant differences between private physicians and dual practitioners (*P* <0.001) and between the latter and the physicians that work in the public sector (*P* = 0.011). Statistically significant differences were only found in the PSPs between the cities of Praia and Bissau (*P* = 0.016).

**Table 2:** **Total hours worked per week, per type of physician**

Country or city	Type of doctors	Mean	N	Std. deviation	Median
Praia	Public	64.12	39	37.8312	57.0
	Private	39.29	14	14.2109	42.8
	Dual practice	68.56	56	24.6138	60.0
	Total	63.21	109	30.4262	57.0
Maputo	Public	61.13	53	34.8456	52.5
	Private	57.93	7	25.6684	48.0
	Dual practice	75.55	64	58.6883	61.8
	Total	68.39	124	48.6334	58.0
Bissau	Public	60.23	46	18.3526	55.5
	Private	58.14	14	16.3559	55.5
	Dual practice	67.52	33	23.4189	62.0
	Total	62.50	93	20.1942	57.0
Total*	Public	61.67	138	31.1607	56.0_a_
	Private	50.56	35	19.5695	49.0_a_
	Dual practice	71.26	153	42.1393	62.0_b_
	Total	64.98	326	36.4069	57.0

*Cells not sharing the same subscript indicate significantly median differences at 5% significance level in a two-sided Kruskal-Wallis test for the equality of the medians of working hours among Private, Public, and Dual practitioners.

The median monthly income of private-only physicians included in the survey was USD 4,405.47 at 2013 exchange rates, with substantial variations across the three locations. Median income among physicians in the public sector was significantly lower than that of physicians in the private sector (*P* <0.001) and dual practice (*P* <0.001), but income differences between private-only and dual practice physicians were not statistically significant (Additional file 2: Table S2 in the statistical annex).

**Figure 2:**
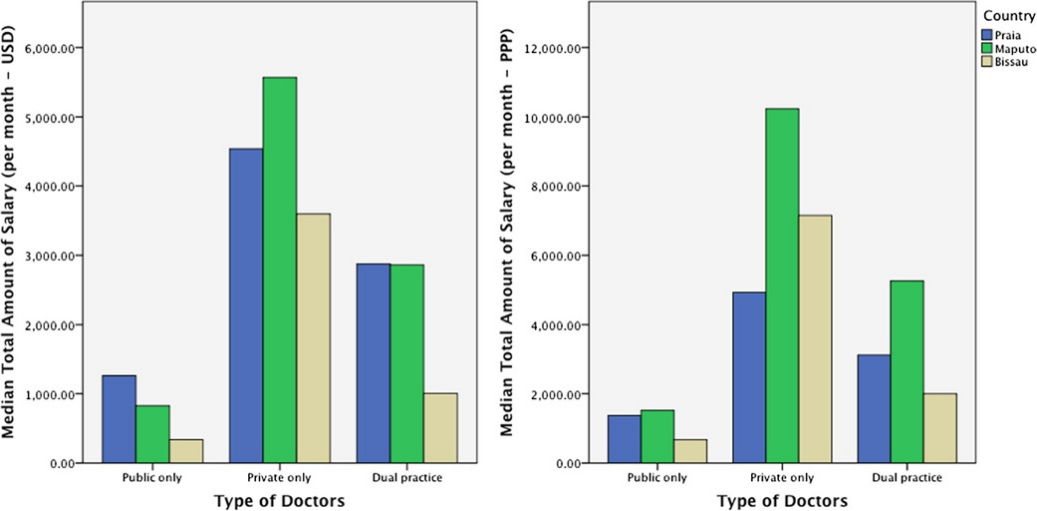
**Median monthly income per location and type of physician.**

Private-only physicians in Maputo were calculated to make the highest monthly revenues (USD 5,318), with public-only physicians earning the lowest (USD 481). Such variations widened when considering purchasing power parity USD (Figure 2).

### Physicians’ motivations to work in the private sector

A consistent set of positive as well as negative motivations for working in the private sector emerged from interviews and surveys, highlighting key distinctions between private-only physicians and dual practitioners. For those working exclusively in the private sector the motivations were higher earnings, autonomy, and flexibility of working hours. For dual practitioners, the main motivations were opportunities to increase income, to consolidate professional reputation, and to take advantage of the complementarities between the two job modalities.

*“I am the boss of my own business; if I decide I don’t want to work tomorrow all I have to do is cancel my appointments”.* Private-only physician from Praia

As to the negative aspects of working in the private sector, PSPs reported private practice’s lack of variety and a less interesting case-mix, as well as the perceived lack of professional collaboration with their peers. For dual practitioners, long working hours, the necessity to work in multiple settings to secure enough patients, and the conflicts of interests resulting from the blurred boundaries between public and private services, were mentioned as downsides of private sector work.

*“This is a very solitary job; the other physicians are no longer colleagues, they are competitors!”.* Private-only physician from Praia.

In the three locations, interviewees concurred that the work carried out in the private sector is generally less sophisticated than in the public, mostly revolving around outpatient visits, and less reliant on cooperation and advice from peers. This was attributed to the limited development of private services especially in Praia and Bissau.

*“All you see in the private sector is kids with a cold. You don’t meet with your colleagues to discuss cases, you don’t get training. And if you run into a complicated diagnosis you refer it back to the public hospital”.* Specialist from the Agostinho Neto Central Hospital, Praia*.*

When asked about the reasons for engaging with the private sector, answers from the survey were broadly consistent with the qualitative findings, with most physicians reporting “increasing income” as the main factor for practicing in the private sector (95.5% responding important or very important), followed by “being able to decide my workload” (56.4%) and “having time to work in both sectors” (12.3%). No association was found between type of employment and the answer “having time to work on both sectors” (*P* = 0.415, χ^2^ test) (See Additional file 2: Table S1 in the statistical annex).

### Personal trajectories of private-only physicians

The qualitative interviews allowed the identification of specific typologies of physicians dedicated exclusively to private practice: the NGO worker, the foreign physician with minimum links with national health authorities, and the senior physician who left the national health system; the latter was the dominant type in Bissau and Maputo. Some of the private-only physicians interviewed reported having been originally trained by the public system and having worked as civil servants, only to leave the National Healthcare Service (NHS) at a later stage, attracted by higher salaries and better working conditions, trading the benefits of long-term but poorly paid public jobs for shorter-term but better rewarded positions.

Interviews showed that, while in Guinea Bissau and Cape Verde leaving the NHS for private jobs is a fairly established practice, it is only a recent phenomenon in Mozambique, following the deterioration of public sector working conditions and the dramatic increase in availability of medical positions in international NGOs.

*“*[…] *it did not use to happen before. Before we were treated differently; perhaps we did not have a high salary, but we were the Ministry of Health, and had several perks.* […] *we had a car, fuel, a telephone and a house. Very recently such perks started disappearing, and one starts being more vulnerable to the offers from private institutions”.* Senior NGO physicians from Maputo.

The foreign physicians interviewed commented that Portuguese-speaking African countries have traditionally benefitted from ideologically-motivated foreign physicians’ technical assistance (*cooperantes)*, especially from Cuba and the former communist countries, but also from comparatively richer African countries such as Nigeria, Senegal, Tanzania, and South Africa. These physicians were reported to have weak ties with the public sector and with National Medical Associations, operated underground and with a business model typical of informal services based on a large volume of patients, little regulation, and low-prices.

*“In the morning I usually see 70–80 patients, then 30–35 in the afternoon. I charge 3000 CFAs if it’s somebody older than 15, but only if he’s a national; now, foreign traders, the UN, diplomats, the* [price of an] *outpatient visit ranges between 5000 and 15000. Foreigners…”.* Private-only physician from Bissau.

Despite their weak links with the national regulatory bodies and erratic compliance with the quality standards of the profession, these private-only physicians were reported to be responding fittingly to the demand for health care services from patients from the lower segment of the market. Some of these physicians saw themselves as competing with public facilities for the lower-end of the market and offering products of similar quality and prices. Some of them even reported having offered their services to the Ministry of Health with the objective of accessing public sector equipment, infrastructure, and clientele.

Finally, senior national physicians who abandoned the public sector to embrace a more entrepreneurial medical activity said that they were the victims of the evolution of the healthcare system from a centralized NHS to a more democratic model integrating public and private services. All were trained as public physicians, and started providing private services within public facilities before the legalization of the private practice; they declared they would have continued to do so but were either dissuaded by the responsible regulatory authority or it became too cumbersome for them to allocate enough working hours to both private and public practices. Other interviewees suggested that such physicians were asked to leave the public as they were taking advantage of the public infrastructure and equipment for their own private benefit. Either way, this type of private-only physicians declared that they would have preferred to keep their public job and conduct their private practice on the side, but felt that the regulatory authorities were “persecuting” private sector-minded physicians, making it hard to perform their private business.

*“There are operation theaters here on the island that are not even switched on in the afternoon, but they* [the government] *would not leave us use them* […]*. We private physicians are widely seen as the ones eating all the meat and leaving only the bones for the public sector physicians; it’s like having a cement roof constantly put on you!”.* Private-only physician from Praia.

Being assigned to a rural facility with no opportunities for private sector services was considered another cause for leaving the public sector, with some younger newly graduated physicians reporting not having even started working for the public sector because of this.

## Discussion

This study aimed at improving the current understanding of characteristics, motivations, choices, and practices of physicians working exclusively in the private sector. The quantitative and qualitative evidence from surveys and interviews in the three African cities show that relatively few physicians work exclusively for the private sector, that they are older and work shorter hours than their public sector and dual practice peers, and that work autonomy and flexibility are the key motivations at the base of their choice to dedicate exclusively to the private sector, since earnings are not significantly different from those of dual practice physicians.

Our study’s methodological approach is affected by some limitations. Firstly, we classified physicians in three categories following their formal employment status. However, recent research has shown that the current definition of dual employment is too narrow and should be expanded to include those public sector physicians offering special private services within public facilities [24, 28]. Secondly, we calculated private sector earnings by multiplying medical acts performed in the private sector by their market prices; on the one hand, such an approach does not separate between physician revenues, profits, and costs. On the other, it may be underestimating private sector earnings as other non-clinical income-generating activities are excluded from the above calculations.

We found that only a limited proportion of physicians in the three locations work exclusively for the private sector (11.2%) with substantial differences across cities, with a majority engaging in private services as dual practitioners, which is broadly consistent with the existing literature [4, 29]. Previous studies have shown how physicians in Maputo enjoy comparatively more opportunities to engage in multiple professional activities [24, 15], which could help explain why these may be more reluctant to abandon the public sector than their colleagues in Praia and Bissau. It is also likely that in Praia, as markets evolve and a clearer distinction between public and private healthcare services is sought, an increasing number of physicians find it more efficient to dedicate exclusively to private practice [30]. On the other hand, the collapse of the public sector health system and the presence of a large informal sector where physicians can operate with few restrictions and minimum cost [31], could offer an explanation for the high proportion of physicians with no ties to the public sector in Bissau.

Our data show that private-only physicians are older and work less hours than their public sector and dual practice peers, which is consistent with the hypothesis that, as they pass the peak of their careers, physicians may be less inclined to engage in multiple professional activities, opting for more flexible and less demanding private sector work [32]. This would also be supported by our qualitative findings on motivations and career trajectories showing that independence and flexibility are the job characteristics private-only physicians value the most. Also a hypothesis could be made that, as physicians grow older, the balance of preference between work and leisure opportunities shifts towards the latter [33]. This would have implications for the success of policies aimed at retaining or attracting back these experienced professionals into public sector jobs by paying higher salaries.

We show that private-only physicians’ earnings could be between four (Praia) and ten times (Bissau) higher than public sector physicians’ wages. However, our data do not show significant differences with dual practitioners’ earnings, and this calculation does not even take into account the tax-free payments these receive from the private services they often offer inside public facilities [24, 26]. This would lend credibility to the hypothesis that increased earning opportunities do not play such a determinant role in physicians’ decisions to abandon the public sector as previously thought, as dual practice seems to be able to guarantee the same level of income. If this contradicts the conventional wisdom that physicians predominantly turn to the private sector for economic reasons [14], this interpretation is consistent with the literature on physicians’ preferences for job characteristics in high-income countries [17, 20].

Our qualitative evidence shows that many private-only physicians who left the NHS claim they would have preferred to keep a professional relation with the public sector. This is consistent with the literature on physicians’ motivation suggesting that, despite the poor conditions offered, public sector work still retains much attraction because of its financial security, the professional values attached to the public sector [13, 18], the more diverse (and probably more interesting) case-mix of patients, and the opportunities for training and for establishing reputation and a professional network [34]. If managed carefully, these positives could be used to the advantage of those strategies aimed at retaining physicians into the public service.

## Conclusions

Physicians working exclusively for the private sector in LMICs have received little attention in the literature, which is remarkable given the interest that private provision of health care services is attracting. We conducted a secondary analysis of primary data from physician interviews and surveys from three African capital cities to deepen the understanding of private-only physicians’ characteristics, time allocation across professional activities, revenues, and motivations.

In comparison to their public sector and dual practice peers, our analysis showed that private-only physicians are predominantly older professionals, working fewer hours, and dedicating more time to managerial functions, and these factors seem to be at the root of their preference for private sector employment. This group appears to include physicians working in the more informal sector and those who parted company with the NHS at a later stage of their career for personal reasons. The study shows the importance of understanding the relation between health professionals’ characteristics, motivations, and practice patterns to develop effective policies to regulate the market of health professionals and achieve universal access to medical services in LMICs.

## Electronic supplementary material

Additional file 1: **Survey on physicians’ dual practice in Portuguese-speaking African countries.** (DOC 292 KB)

Additional file 2: **Statistical annex.** (DOC 62 KB)

## Abbreviations

LMICs: Low- and middle-income countries
NGOs: Non-governmental and faith-based organisations
NHS: National Healthcare Service
PSP: Private sector physicians
USD: United Stated Dollars.
