# Supplementary material for: Why do some physicians in Portuguese-speaking African countries work exclusively for the private sector? Findings from a mixed-methods study
Source: Hum Resour Health. 2014 Sep 11;12:51. doi: 10.1186/1478-4491-12-51 (PMC4167285; doi:10.1186/1478-4491-12-51)
Supplement: Supplementary file 1 — Additional file 1: Survey on physicians’ dual practice in Portuguese-speaking African countries. (DOC 292 KB) [file 12960_2014_446_MOESM1_ESM.doc]

**Additional file 1: SURVEY ON PHYSICIANS’ DUAL PRACTICE IN PORTUGUESE-SPEAKING AFRICAN COUNTRIES**

**A. GENERAL CHARACTERISTICS**

**1. Age**: _____ years

**2. Sex**

|  | 0. Male |
| --- | --- |
|  |  |
|  | 1. Female |
|  |  |

**3. Civil status**

|  | 0. Married |
| --- | --- |
|  |  |
|  | 1. Unmarried |
|  |  |

**NOTE: please mark whether the respondent is currently living with partner**

**4. Have you got dependents (for example, spouse or children who depend on your income for subsistence)?**

|  | 0. No |
| --- | --- |
|  |  |
|  | 1. Yes. How many? ______ |
|  |  |

**5. Have you got any physician in your family?**

|  | 0. No |
| --- | --- |
|  |  |
|  | 1. Yes. |
|  |  |

**6. For how long have you worked as a medical doctor in Praia/Bissau/Maputo?** _____ years

**NOTE:** if respondent refers less than a year, write 0.

**7. Do you currently work as physician outside Praia/Bissau/Maputo too**?

|  | 0. No |
| --- | --- |
|  |  |
|  | 1. Yes. |
|  |  |

**8. Do you hold any specialisation in medicine**?

|  | 0. No |
| --- | --- |
|  |  |
|  | 1. Yes. Which one? ______________________________________________________________ |
|  |  |

**NOTA**: Please write exactly as the specialisation is referred.

**B. WORKLOAD IDENTIFICATION AND CALCULATION**

**9. WHAT IS YOUR TYPICAL Weekly WORK LOAD LIKE? Please fill in the table below identifying all income-generating activities (e.g. outpatient visits, inpatient visits, teaching, etc.)**

| **Hours** | **Monday** | | **Tuesday** | | **Wednesday** | | **Thursday** | | **Friday** | | **Saturday** | | **Sunday** | |
| --- | --- | --- | --- | --- | --- | --- | --- | --- | --- | --- | --- | --- | --- | --- |
|  | **Activity** | **Total number of patients seen** | **Activity** | **Total number of patients seen** | **Activity** | **Total number of patients seen** | **Activity** | **Total number of patients seen** | **Activity** | **Total number of patients seen** | **Activity** | **Total number of patients seen** | **Activity** | **Total number of patients seen** |
| 6-7 |  |  |  |  |  |  |  |  |  |  |  |  |  |  |
| 7-8 |  |  |  |  |  |  |  |  |  |  |  |  |  |  |
| 8-9 |  |  |  |  |  |  |  |  |  |  |  |  |  |  |
| 9-10 |  |  |  |  |  |  |  |  |  |  |  |  |  |  |
| 10-11 |  |  |  |  |  |  |  |  |  |  |  |  |  |  |
| 11-12 |  |  |  |  |  |  |  |  |  |  |  |  |  |  |
| 12-13 |  |  |  |  |  |  |  |  |  |  |  |  |  |  |
| 13-14 |  |  |  |  |  |  |  |  |  |  |  |  |  |  |
| 14-15 |  |  |  |  |  |  |  |  |  |  |  |  |  |  |
| 15-16 |  |  |  |  |  |  |  |  |  |  |  |  |  |  |
| 16-17 |  |  |  |  |  |  |  |  |  |  |  |  |  |  |
| 17-18 |  |  |  |  |  |  |  |  |  |  |  |  |  |  |
| 18-19 |  |  |  |  |  |  |  |  |  |  |  |  |  |  |
| 19-20 |  |  |  |  |  |  |  |  |  |  |  |  |  |  |
| 20-21 |  |  |  |  |  |  |  |  |  |  |  |  |  |  |
| 21-22 |  |  |  |  |  |  |  |  |  |  |  |  |  |  |
| 22-23 |  |  |  |  |  |  |  |  |  |  |  |  |  |  |
| 23-24 |  |  |  |  |  |  |  |  |  |  |  |  |  |  |
| 0-1 |  |  |  |  |  |  |  |  |  |  |  |  |  |  |
| 1-2 |  |  |  |  |  |  |  |  |  |  |  |  |  |  |
| 2-3 |  |  |  |  |  |  |  |  |  |  |  |  |  |  |
| 3-4 |  |  |  |  |  |  |  |  |  |  |  |  |  |  |
| 4-5 |  |  |  |  |  |  |  |  |  |  |  |  |  |  |
| 5-6 |  |  |  |  |  |  |  |  |  |  |  |  |  |  |

**NOTE:** The total number of patients per hour spent in each activity need to be imputed.

**C. PUBLIC SECTOR (CLINICAL)**

**10. Are you currently employed in the NHS**?

|  | 0. No. **Please move on to section D. PRIVATE SECTOR (CLINICAL)** |
| --- | --- |
|  |  |
|  | 1. Yes. |
|  |  |

**11. Do you currently do clinical work for the NHS (e.g. outpatient visits)?**

|  | 0. No. **Please move on to section D. PRIVATE SECTOR (CLINICAL)** |
| --- | --- |
|  |  |
|  | 1. Yes. |
|  |  |

**12. At what level of care do you currently work? Please tick all the options applicable.**

|  | 1. Health centre |
| --- | --- |
|  |  |
|  | 2. Central hospital |
|  |  |
|  | 3. Other hospital |
|  |  |
|  | 4. Other. Which one?_________________________________ |
|  |  |

**13. Below are listed some of the main reasons physicians give for working in the public sector. Please state your agreement with such options, specifying whether each of them are: 1. Not important; 2. Somewhat important; 3. Important, and; 4. Very important.**

| a. There are not enough opportunities in the private sector. | 1. Not important | 2. Somewhat important | 3. Important | 4 Very important |
| --- | --- | --- | --- | --- |
|  |  |  |  |  |
| b. To gain experience. | 1. Not important | 2. Somewhat important | 3. Important | 4 Very important |
|  |  |  |  |  |
| c. Work is rewarding. | 1. Not important | 2. Somewhat important | 3. Important | 4 Very important |
|  |  |  |  |  |
| d. It gives access to further training and conferences. | 1. Not important | 2. Somewhat important | 3. Important | 4 Very important |
|  |  |  |  |  |
| e. It provides patients for private sector practice. | 1. Not important | 2. Somewhat important | 3. Important | 4 Very important |
|  |  |  |  |  |
| f. It helps build up your professional reputation. | 1. Not important | 2. Somewhat important | 3. Important | 4 Very important |
|  |  |  |  |  |
| g. Working in the public helps providing better care to your private sector patients. | 1. Not important | 2. Somewhat important | 3. Important | 4 Very important |
|  |  |  |  |  |
| h. Public sector jobs are more secure than private sector ones. | 1. Not important | 2. Somewhat important | 3. Important | 4 Very important |
|  |  |  |  |  |
| i. It provides for a pension scheme. | 1. Not important | 2. Somewhat important | 3. Important | 4 Very important |
|  |  |  |  |  |
| j. It provides medical assistance | 1. Not important | 2. Somewhat important | 3. Important | 4 Very important |

**13.1. Are there further reasons for working in the public beyond those mentioned above?**

|  | 0. No. |
| --- | --- |
|  |  |
|  | 1. Yes. Which ones?________________________________________________________ |
|  |  |

**14. What is your take away salary in the public sector?** ________CVE / CFA /MTn

**15. For your public sector work, do you get any other salary** (e.g. revenues from special services)?

|  | 0. No |
| --- | --- |
|  |  |
|  | 1. Yes |
|  |  |

NOTE: Special services are defined as those paid out-of-pocket by patients or by a health insurance

**16. Below are listed some of the main reasons physicians give for engaging in special services/clinics. Please state your agreement with such options, specifying whether each of them are: 1. Not important; 2. Somewhat important; 3. Important, and; 4. Very important.**

| a. Gain more clients | 1. Not important | 2. Somewhat important | 3. Important | 4 Very important |
| --- | --- | --- | --- | --- |
|  |  |  |  |  |
| b. Gain an extra income | 1. Not important | 2. Somewhat important | 3. Important | 4 Very important |
|  |  |  |  |  |
| c. In comparison to private practice, it does not require an initial investment | 1. Not important | 2. Somewhat important | 3. Important | 4 Very important |
|  |  |  |  |  |
| d. It is close to your public sector place of work | 1. Not important | 2. Somewhat important | 3. Important | 4 Very important |
|  |  |  |  |  |
| e. It is possible to do it in the morning | 1. Not important | 2. Somewhat important | 3. Important | 4 Very important |
|  |  |  |  |  |

**16.1. Are there further reasons for working in the public beyond those mentioned above?**

|  | 0. No. |
| --- | --- |
|  |  |
|  | 1. Yes. Which?_____________________________________________________________ |

**D. PRIVATE SECTOR (CLINICAL)**

**17. Do you currently work as physician in the private sector?** (by ‘private sector’ it is here intended clinical services not paid by the state)**?**

|  | 0. No. **Please proceed to section E. Regulation** |
| --- | --- |
|  |  |
|  | 1. Yes. |
|  |  |

**18. In what capacity?** Please tick all applicable options.

|  | 1. As a clinical staff. |
| --- | --- |
|  |  |
|  | 2. As a manager. |
|  |  |
|  | 3. As a consultant. |
|  |  |
|  | 4. Other. Please specify which._______________________________________________ |
|  |  |

**19. What sort of private sector institution do you work for?** Please tick all applicable options.

|  | 1. Your own private practice. |
| --- | --- |
|  |  |
|  | 2. Private practice owned by colleagues. |
|  |  |
|  | 3. Private clinic. |
|  |  |
|  | 4. Private hospital. |
|  |  |
|  | 5. House visits. |
|  |  |
|  | 6. Other. Please specify which. |
|  |  |

**20. How many hours do you work on average per week in the private sector?**___ Hours.

**21. For what reason you do not happen to work more hours in the private service? Below are listed some of the main reasons physicians give for not spending more hours in the private sector. Please state your agreement with such options, specifying whether: 1. Do not agree; 2. Somewhat agree; 3. Agree, and; 4. Agree very much.**

| a. There are not enough patients in the private sector. | 1. Do not agree | 2. Somewhat agree | 3. Agree | 4. Agree very much |
| --- | --- | --- | --- | --- |
|  |  |  |  |  |
| b. I do not have enough access to private sector opportunities. | 1. Do not agree | 2. Somewhat agree | 3. Agree | 4. Agree very much |
|  |  |  |  |  |
| b. I already work enough hours. | 1. Do not agree | 2. Somewhat agree | 3. Agree | 4. Agree very much |
|  |  |  |  |  |
| c. I have other personal priorities. | 1. Do not agree | 2. Somewhat agree | 3. Agree | 4. Agree very much |
|  |  |  |  |  |
| d. I would like to work more hours, but to do that, I should be looking into other private clinics. | 1. Do not agree | 2. Somewhat agree | 3. Agree | 4. Agree very much |
|  |  |  |  |  |

**21.1. Are there further reasons for working in the private beyond those mentioned above?**

|  | 0. No. |
| --- | --- |
|  |  |
|  | 1. Yes. Please specify._____________________________________________________ |

**22. If you had one extra hour to spend in the private sector, to what activity would you dedicate your extra time? Please tick one option only.**

|  | 1. Outpatient visits. |
| --- | --- |
|  |  |
|  | 2. Inpatient visits. |
|  |  |
|  | 3. High-cost exams (CT scan, echography etc.) |
|  |  |
|  | 4. Low-cost exams (X-rays, lab tests etc.) |
|  |  |
|  | 5. Minor surgery interventions. |
|  |  |
|  | 6. Major surgery interventions. |
|  |  |
|  | 6. Other. Please specify. |
|  |  |

**22.1 How many extra patients do you think you would be able to see in such extra hour?**

| 1. Outpatient visits. | _______patients |
| --- | --- |
|  |  |
| 2. Inpatient visits. | _______patients |
|  |  |
| 3. High-cost exams (CT scan, echography etc.) | _______patients |
|  |  |
| 4. Low-cost exams (X-rays, lab tests etc.) | _______patients |
|  |  |
| 5. Minor surgery interventions. | _______patients |
|  |  |
| 6. Major surgery interventions. | _______patients |
|  |  |
| 7. Other. Please specify. | _______patients |
|  |  |

**NOTE:** For those patients not wishing to work extra hours, please mark not applicable **(NA)**. If respondent does not know, please mark DK.

**23. Below are listed some of the main reasons physicians give for wishing to work in the private sector. Please state your agreement with such options, specifying whether: 1. Not important; 2. Somewhat important; 3. Important, and; 4. Very important.**

| a. Increase your income | 1. Not important | 2. Somewhat important | 3. Important | 4 Very important |
| --- | --- | --- | --- | --- |
|  |  |  |  |  |
| b. Have enough time to work both in public and private | 1. Not important | 2. Somewhat important | 3. Important | 4 Very important |
|  |  |  |  |  |
| c. Being able to decide your own workload. | 1. Not important | 2. Somewhat important | 3. Important | 4 Very important |
|  |  |  |  |  |

**23.1. Are there further reasons for working in the public beyond those mentioned above?**

|  | 0. No. |
| --- | --- |
|  |  |
|  | 1. Yes. Please specify. ______________________________________________________ |

**24. What is the average price of the following medical acts in the private practice where you work?**

| a. Outpatient visit (per unit) | __________ CVE / CFA /MTn per visit |
| --- | --- |
|  |  |
| b. In patient visit per unit | __________ CVE / CFA /MTn per occupied bed-day |
|  |  |
| c. High-cost exams | __________ CVE / CFA /MTn per exam |
|  |  |
| d. Lo-cost exams | __________ CVE / CFA /MTn per exam |
|  |  |
| e. Minor surgical interventions. | __________ CVE / CFA /MTn per intervention |
|  |  |
| f. Minor surgical interventions. | __________ CVE / CFA /MTn per intervention |
|  |  |
| g. Home visits. | __________ CVE / CFA /MTn per visit |

**E. REGULATION**

**25. In your opinion, physician dual practice should be regulated?** (e.g. laying rules on what physicians can engage in dual practice, and in what terms)

|  | 0. No. **Please proceed to question Nº 28.** |
| --- | --- |
|  |  |
|  | 1. Yes. |
|  |  |

**26. Below are listed some of the main reasons why physicians think dual practice should be regulated. Please state your agreement with such options, specifying whether each of them are: 1. Not important; 2. Somewhat important; 3. Important, and; 4. Very important.**

| a. This is a good way to expand medical services to population. | 1. Not important | 2. Somewhat important | 3. Important | 4 Very important |
| --- | --- | --- | --- | --- |
|  |  |  |  |  |
| b. It is a good way to increase physicians’ income. | 1. Not important | 2. Somewhat important | 3. Important | 4 Very important |
|  |  |  |  |  |
| c. The practice is currently only accessible to some physicians and discriminate others. | 1. Not important | 2. Somewhat important | 3. Important | 4 Very important |
|  |  |  |  |  |
| d. Public sector activities can suffer from lack of dual practice regulation. | 1. Not important | 2. Somewhat important | 3. Important | 4 Very important |
|  |  |  |  |  |

**26.1. Are there further reasons for working in the public beyond those mentioned above?**

|  | 0. No. |
| --- | --- |
|  |  |
|  | 1. Yes. Please specify.___________________________ __________________________ |

**27. In your opinion, what institution should regulate the practice? (mark all applicable)**

|  | 1. The Government |
| --- | --- |
|  |  |
|  | 2. Ministry of Health |
|  |  |
|  | 3. The Medical Association |
|  |  |
|  | 4. Other. Please specify __________________________________ |
|  |  |

**28. In your opinion, physician dual practice in the last decade has become:** (please mark only one option)

|  | 1. More regulated |
| --- | --- |
|  |  |
|  | 2. Less regulated |
|  |  |
|  | 3. There has not been any major change |
|  |  |

**29. What consequence the recent changes in dual practice regulation in your country have had in your specific case?** (Please mark only one option)

|  | 1. Nothing has changed |
| --- | --- |
|  |  |
|  | 2. I am spending more time in the public sector |
|  |  |
|  | 3. I am spending more time in the private sector |
|  |  |
|  | 4. I changed job |
|  |  |
|  | 5. I changed work location |
|  |  |
|  | 6. I asked to change job/location |
|  |  |
|  | 7. Other (please specify) __________________________________ |
|  |  |

**30. Below are listed some regulatory options for the PUBLIC SECTOR. Please state your agreement with such options, specifying whether: 1. Do not agree; 2. Somewhat agree; 3. Agree, and; 4. Agree very much.**

| a. Less regulation of public sector medical activities | 1. Do not agree | 2. Somewhat agree | 3. Agree | 4. Agree very much |
| --- | --- | --- | --- | --- |
|  |  |  |  |  |
| b. More control on hours spent/worked in public sector | 1. Do not agree | 2. Somewhat agree | 3. Agree | 4. Agree very much |
|  |  |  |  |  |
| c. General ban on special services in public institutions | 1. Do not agree | 2. Somewhat agree | 3. Agree | 4. Agree very much |
|  |  |  |  |  |
| d. Legalisation of special services in public institutions | 1. Do not agree | 2. Somewhat agree | 3. Agree | 4. Agree very much |
|  |  |  |  |  |
| e. More control of revenues generated through special services in public institutions | 1. Do not agree | 2. Somewhat agree | 3. Agree | 4. Agree very much |
|  |  |  |  |  |

**30.1. Are there further reasons for working in the public beyond those mentioned above?**

|  | 0. No. |
| --- | --- |
|  |  |
|  | 1. Yes. Please specify. _________________________________________________ |

**31. Below are listed some regulatory options for the PRIVATE SECTOR. Please state your agreement with such options, specifying whether: 1. Do not agree; 2. Somewhat agree; 3. Agree, and; 4. Agree very much.**

| a. Less regulation in private sector medical activities. | 1. Do not agree | 2. Somewhat agree | 3. Agree | 4. Agree very much |
| --- | --- | --- | --- | --- |
|  |  |  |  |  |
| b. More control of hours spent in public. | 1. Do not agree | 2. Somewhat agree | 3. Agree | 4. Agree very much |
|  |  |  |  |  |
| c. Improve availability of bank loans to physicians to start private activity | 1. Do not agree | 2. Somewhat agree | 3. Agree | 4. Agree very much |
|  |  |  |  |  |
| d. Government incentives to physicians to start private activity. | 1. Do not agree | 2. Somewhat agree | 3. Agree | 4. Agree very much |
|  |  |  |  |  |
| e. Less tax on private sector revenues. | 1. Do not agree | 2. Somewhat agree | 3. Agree | 4. Agree very much |
|  |  |  |  |  |
| f. Ban of medical special services within public institutions | 1. Do not agree | 2. Somewhat agree | 3. Agree | 4. Agree very much |
|  |  |  |  |  |
| g. Legalisation of medical special services within public institutions | 1. Do not agree | 2. Somewhat agree | 3. Agree | 4. Agree very much |
|  |  |  |  |  |

**31.1. Are there further reasons for working in the public beyond those mentioned above?**

|  | 0. No. |
| --- | --- |
|  |  |
|  | 1. Yes. Please specify. |

**MANY THANKS FOR YOUR COLLABORATION. IF YOU WANT TO LEARN THE RESULTS FROM THE SURVEY, PLEASE INQUIRY WITH THE NATIONAL MEDICAL COUNCIL.**

**F. WORKLOAD TABLE**

| **Sector** | **ACTIVITY** | **Total hours /per week** | **Total patients/per week** |
| --- | --- | --- | --- |
| Public | Outpatient visits |  |  |
|  | In patient visits |  |  |
|  | Supervision of trainees |  |  |
|  | Formal teaching (e.g. Faculty of Medicine etc.) |  |  |
|  | Administration |  |  |
|  | Policy work |  |  |
|  | Teaching and training |  |  |
|  | Emergency and resuscitation |  |  |
|  | Other (please specify) |  |  |
|  |  |  |  |
|  |  |  |  |
| Private | Outpatient visits |  |  |
|  | In patient visits |  |  |
|  | Supervision of trainees |  |  |
|  | Formal teaching (e.g. Faculty of Medicine etc.) |  |  |
|  | Administration |  |  |
|  | Policy work |  |  |
|  | Teaching and training |  |  |
|  | Emergency and resuscitation |  |  |
|  | Other (please specify) |  |  |
