# Supplementary material for: Why do some physicians in Portuguese-speaking African countries work exclusively for the private sector? Findings from a mixed-methods study
Source: Hum Resour Health. 2014 Sep 11;12:51. doi: 10.1186/1478-4491-12-51 (PMC4167285; doi:10.1186/1478-4491-12-51)
Supplement: Supplementary file 2 — Additional file 2: Statistical annex. (DOC 62 KB) [file 12960_2014_446_MOESM2_ESM.doc]

# Annex I: Statistical annex

Table S1: Proportion of physicians agreeing on reasons to practice in the private sector, by type of physicians

| Type of doctors | |  | **Increase income** | | | | |
| --- | --- | --- | --- | --- | --- | --- | --- |
|  |  |  | Not important | Somewhat important | Important | Very important | Total |
|  | Private only | Count | 1 | 3 | 5 | 22 | 31 |
|  |  | Std. Residuals | 2.0 | 2.3 | -0.6 | -0.3 |  |
|  | Dual practitioners | Count | 0 | 2 | 33 | 114 | 49 |
|  |  | Std. Residuals | -0.9 | -1.1 | 0.3 | 0.1 |  |
| Total |  | Count | 1 | 5 | 38 | 136 | 180 |
|  |  |  |  |  |  |  |  |
| Type of doctors | |  | **Be able to decide how many hours to work** | | | | |
|  |  |  | Not important | Somewhat important | Important | Very important | Total |
|  | Private only | Count | 3 | 3 | 19 | 6 | 31 |
|  |  | Std. Residuals | -1.6 | -1.2 | 1.1 | 2.1 |  |
|  | Dual practitioners | Count | 40 | 32 | 67 | 9 | 148 |
|  |  | Std. Residuals | 0.7 | 0.6 | -0.5 | -1 |  |
| Total |  | Count | 43 | 35 | 86 | 15 | 179 |

Table S2: Physicians' mean monthly income, per location and type of physicians (at 2012 USD exchange rate and PPP)

| Country or city | Type of Doctors | N | USD at exchange rates | Std. Deviation | PPP USD | Std. Deviation |
| --- | --- | --- | --- | --- | --- | --- |
| Praia | Public | 27 | 1270.0655 | 245.75269 | 1378.6998 | 266.773 |
|  | Private | 13 | 4649.133 | 2876.5246 | 5046.7939 | 3122.56646 |
|  | Dual practice | 55 | 3234.0118 | 1736.4928 | 3510.6312 | 1885.0227 |
|  | Total | 95 | 2869.4858 | 2013.90414 | 3114.9256 | 2186.16226 |
| Maputo | Public | 29 | 1245.2973 | 1445.73645 | 2286.8473 | 2654.93106 |
|  | Private | 7 | 5318.0009 | 3566.52661 | 9765.9056 | 6549.5217 |
|  | Dual practice | 61 | 3312.9867 | 2467.52216 | 6083.9245 | 4531.32465 |
|  | Total | 97 | 2839.5033 | 2562.84396 | 5214.4258 | 4706.37233 |
| Bissau | Public | 36 | 481.0647 | 605.9173 | 956.6757 | 1204.96543 |
|  | Private | 10 | 2882.3193 | 2050.8478 | 5731.9622 | 4078.44551 |
|  | Dual practice | 31 | 1314.1536 | 1006.22723 | 2613.409 | 2001.04704 |
|  | Total | 77 | 1128.3154 | 1297.75034 | 2243.8393 | 2580.78832 |
| Total | Public | 92 | 953.5187 | 973.01126 | 1499.8238 | 1751.01233 |
|  | Private | 30 | 4216.2643 | 2889.89873 | 6376.3094 | 4666.98692 |
|  | Dual practice | 147 | 2861.9157 | 2114.55449 | 4389.2502 | 3568.68632 |
|  | Total | 269 | 2360.2723 | 2205.45548 | 3622.65 | 3613.04334 |
